# Prospects for nasal delivery of a pharmacologic agent for neuroprotective experimental therapy after prenatal hypoxia

**DOI:** 10.3389/fnsys.2025.1670565

**Published:** 2025-11-10

**Authors:** Igor Belenichev, Olena Aliyeva, Nina Bukhtiyarova, Victor Ryzhenko, Bogdan Burlaka, Kristina Burlaka, Dmytro Skoryna, Pavlo Petakh, Oleksandr Kamyshnyi

**Affiliations:** 1 Department of Pharmacology and Medical Formulation with Course of Normal Physiology, Zaporizhzhia State Medical and Pharmaceutical University, Zaporizhzhia, Ukraine; 2 Department of Histology, Cytology and Embryology, Zaporizhzhia State Medical and Pharmaceutical University, Zaporizhzhia, Ukraine; 3 Department of Clinical Laboratory Diagnostics, Zaporizhzhia State Medical and Pharmaceutical University, Zaporizhzhia, Ukraine; 4 Department of Medical and Pharmaceutical Informatics and Advanced Technologies, Zaporizhzhia State Medical and Pharmaceutical University, Zaporizhzhia, Ukraine; 5 Department of Medicines Technology, Zaporizhzhia State Medical and Pharmaceutical University, Zaporizhzhia, Ukraine; 6 Department of Pharmaceutical, Organic and Bioorganic Chemistry, Zaporizhzhia State Medical and Pharmaceutical University, Zaporizhzhia, Ukraine; 7 Department of Biochemistry and Pharmacology, Uzhhorod National University, Uzhhorod, Ukraine; 8 Department of Microbiology, Virology, and Immunology, I. Horbachevsky Ternopil National Medical University, Ternopil, Ukraine

**Keywords:** prenatal hypoxia, neuroprotection, mitochondrial dysfunction, oxidative stress, nitrosative stress, intranasal delivery, Angiolin gel

## Abstract

Prenatal hypoxia (PH) significantly impacts the central nervous system (CNS) development, often resulting in long-term cognitive, behavioral, and neurological deficits due to oxidative stress, mitochondrial dysfunction, and neuroapoptosis. The brain’s endogenous protective mechanisms are often insufficient under prolonged hypoxia, necessitating the development of novel neuroprotective strategies. This study aimed to evaluate the neuroprotective efficacy of nasal administration of Angiolin gel—a novel pharmacological agent—in experimental model of PH. Chronic intrauterine hypoxia was induced in pregnant rats via sodium nitrite administration. Newborn rats were divided into groups receiving either Angiolin gel intranasally, Piracetam intraperitoneally, or saline (control) for 30 days. Biochemical, morphometric, histoimmunochemical, and neurophysiological methods were employed to assess outcomes. The results demonstrated that PH induced mitochondrial dysfunction, oxidative and nitrosative stress, GABAergic system impairment, and neuroapoptosis, leading to increased neonatal mortality and deficits in cognitive and motor functions. Angiolin gel administration significantly enhanced energy metabolism by restoring mitochondrial enzyme activities (SDH, MDH, and CPK), increasing ATP production, and reducing lactate accumulation. It also normalized GABAergic parameters, increased the activity of antioxidant enzymes (Cu/Zn-SOD, GPX1/4) and decreased nitrosative stress markers (iNOS, nitrotyrosine). Histomorphometric analysis revealed preserved neuronal density and reduced apoptosis in the hippocampus, alongside enhanced Fos/Bcl-2 expression. Behavioral tests demonstrated improved motor activity, memory retention, and exploratory behavior, with a 47% reduction in early mortality. Comparative analysis showed superior efficacy of Angiolin over Piracetam, which exacerbated lactate acidosis. These findings suggest that intranasal administration of Angiolin gel effectively targets multiple pathophysiological pathways triggered by PH, providing robust neuroprotection and promoting functional recovery. Given its favorable safety profile and the non-invasive nature of intranasal delivery, Angiolin gel represents a promising therapeutic approach for mitigating the long-term neurological consequences of prenatal hypoxia and warrants further clinical investigation in neonatal and pediatric neurology.

## 1 Introduction

According to data from researchers and clinicians in the European Union, the incidence of registered cases of prenatal hypoxia (PH) is approximately 2.8% (Zimbeck et al., 2009; Fawole and Hofmeyr, 2012; Wang et al., 2021; Dev et al., 2022; United States Department of Health and Human Services (US DHHS), Centers for Disease Control and Prevention (CDC), National Center for Health Statistics (NCHS), 2021; Gessesse et al., 2024). The etiological factors contributing to PH are well-documented and include adverse environmental conditions, maternal illnesses (e.g., infections, anemia, diabetes mellitus, obesity, pulmonary and cardiac insufficiency), substance abuse (alcohol, narcotics, psychostimulants, smoking), and certain medications. Other contributing factors include maternal anemia, hemoglobinopathies, placental insufficiency, and umbilical cord compression (Bhatt et al., 2014; Capra et al., 2013; Kumar et al., 2022; Ouédraogo et al., 2013; Kankowski et al., 2022; Driscoll et al., 2018; Institute of Medicine (US) Committee on Nutritional Status During Pregnancy and Lactation, 1990).

Although the fetal brain, a primary target of hypoxic damage, possesses intrinsic protective mechanisms, these defenses may be insufficient under conditions of prolonged hypoxia. Consequently, the brain cells are undergoing destructive damage (Giussani, 2016; Ducsay et al., 2018). PH decreases the number of neurons and synaptic density in the hippocampus, alters the release and reuptake of neurotransmitters and leads to the development of transmitter autocoidosis, increased production of ROS, oxidative stress and neuroapoptosis. ROS overproduction in consequence of PH occurs by mitochodria due to the formation of mitochondrial dysfunction and energy deficiency. The damaged mitochondrion is a source of not only ROS, but also proapotic and proinflammatory factors (Allen and Brandon, 2011; Nicosia et al., 2024; Piña-Crespo et al., 2014; da Silva et al., 2021; Bertozzi et al., 2024; Jomova et al., 2023).

Another source of ROS is the consequences of glutamate excitotoxicity and activation of nNOS and iNOS (Armada-Moreira et al., 2020; Gillessen et al., 2002; Wu et al., 2025). Oxidative stress results in oxidative modification of proteins and nucleic acids, leading to receptor desensitization, disruption of reverberation of nerve impulse and ionic homeostasis, and ultimately, dysfunction of higher functions of the central nervous system (CNS; Salim, 2017; Trofin et al., 2025; Kim et al., 2024; Wilson et al., 2022; Krishnamurthy et al., 2024). Offspring exposed to PH often exhibit impaired motor and exploratory activity, learning and memory deficits, and emotional disturbances such as aggression, anxiety, and fear (Golan and Huleihel, 2006; Aliyeva et al., 2025; Miller et al., 2016; Monk et al., 2013). Furthermore, PH can adversely affect CNS development, increasing the risk of long-term behavioral and cognitive impairments and predisposing individuals to neurodegenerative diseases such as Parkinson’s and Alzheimer’s (Wang et al., 2021; Amartumur et al., 2024; Guo et al., 2022; Orzeł et al., 2023).

We hypothesized that enhancing endogenous heat shock protein 70 (HSP70) through pharmacological modulation during hypoxic neurodegeneration could offer a novel neuroprotective strategy for brain disorders of different genesis (Belenichev et al., 2015a, 2025). These studies have expanded our understanding of HSP70’s role in modulating neuroapoptotic signaling pathways and have elucidated its involvement in endogenous neuroprotective mechanisms. We have formed systemic concept about the role of GSH/HSP70—mechanisms of endogenous neuroprotection in the prevention of pathobiochemical, morphofunctional changes of brain cells under ischemia, hypoxia, including PH and experimentally substantiated new target links for neuroprotection. New data on the mechanisms of activation and regulation of GSH/HSP 70-dependent mechanisms of endogenous neuroprotection and experimental substantiation of the use of modulators of this system as promising neuroprotectors were obtained (Belenichev et al., 2023b; Belenichev et al., 2024a).

In experimental studies, we demonstrated that administration of HSP70 modulators—including Cerebrocurin, Angiolin, Tamoxifen, Glutoredoxin, Thiotriazolin, HSF-1 (heat shock factor protein 1), as well as Mildronate, Mexidol in the first 30 days of life to offspring after PH resulted in reduced early mortality and improved motor, exploratory, and cognitive functions in offspring following PH. These effects were associated with increased expression of HSP70 and hypoxia-inducible factor 1 (HIF-1) in the brain. Among the tested compounds, Angiolin emerged as the most effective neuroprotective agent. We have substantiated the expediency of advanced research and developed an intranasal dosage form with Angiolin with significant neuroprotective properties and a high safety profile (Aliyeva et al., 2025; Belenichev et al., 2024a). These findings underscore the relevance and promise of advanced studies of intranasal gel with Angiolin as a neuroprotective agent after PH.

The aim of the study: To conduct a comprehensive evaluation of the neuroprotective effects of intranasally administered Angiolin gel in an experimental model of PH.

## 2 Materials and methods

### 2.1 Pharmaco-technological methods

#### 2.1.1 Formulation design

The formulation of the Angiolin nasal gel was developed within the laboratory of the Department of Drug Technology of Zaporizhzhia State Medical and Pharmaceutical University. The active pharmaceutical ingredient employed was Angiolin [(S)-2,6-diaminohexanoic acid 3-methyl-1,2,4-triazolyl-5-thioacetate], provided by the Scientific and Technological Complex “Institute of Monocrystals” of the National Academy of Sciences of Ukraine. The excipients incorporated in the formulation included D-panthenol, sodium salt of carboxymethyl cellulose (CMC), Tween-80, benzalkonium chloride, and purified water. All active and excipient substances used in the experiments conformed to pharmaceutical-grade standards and were sourced from SPA “SINBIAS,” “Istok-Plus” LLC, and SPA “Pharmatron.” The selection and proportion of excipients in the Angiolin nasal gel were established based on prior research findings (Belenichev et al., 2024b; Table 1).

**Table 1:** Pharmaceutical formulation of nasal gel with angiolin.

Ingredients	Quantity (g)
Angiolin	1.0
Sodium CMC	1.5
D-panthenol	1
Benzalkonium chloride	0.02
Tween-80	1.2
Purified water	Up to 100

The optimized manufacturing process for the Angiolin nasal gel formulation involves the sequential preparation and combination of two distinct mixtures, designated as Mixture A and Mixture B, to yield a final gel volume of 100 g.

Preparation of Mixture A: Precisely 47.64 mL of purified water is combined with 1.5 g of sodium carboxymethylcellulose (CMC). This mixture was heated to 70–80 °C and maintained at that temperature for 1 h to allow adequate swelling of the polymer. Following the heating phase, the mixture is allowed to cool to ambient temperature (approximately 25 °C), after which 1.0 g of D-panthenol is incorporated under continuous low-speed magnetic stirring to ensure uniform dispersion.

Preparation of Mixture B: An additional 47.64 mL of purified water is used to dissolve 1.0 g of Angiolin and 1.2 g of Tween-80, utilizing magnetic stirring to achieve a clear, homogeneous solution with a characteristic pale-yellow hue attributable to Tween-80. Subsequently, 0.02 g of benzalkonium chloride is introduced under constant agitation to ensure thorough incorporation.

Final Step: The prepared Mixture B is gradually introduced into Mixture A under continuous stirring, resulting in the formation of a uniform nasal gel with a consistent yellowish appearance.

#### 2.1.2 Pharmaceutical characterization of Angiolin nasal gel

##### 2.1.2.1 Target product profile and critical quality attributes

Dosage form: intranasal mucoadhesive gel with neuroprotective agent angiolin (1%).

(1) Target CQAs and specifications (at the design stage)

pH: 5.5–6.5 (physiological range of the nasal mucosa; minimization of irritation and optimal tolerability; Patharapankal et al., 2024).

Rheology (27–32 °C): viscosity and thixotropy ensuring retention on the mucosa without interfering with mucociliary clearance.

Active substance release: complete kinetic curve at model environment temperature (27–32 °C).

Stability: control of pH, viscosity, active ingredient content.

(2) Composition and rational selection of excipients

Optimized formula (100 g): Angiolin 1.0; Na-CMC 1.5; D-panthenol 1.0; Tween-80 1.2; benzalkonium chloride 0.02; purified water—up to 100. The formula was obtained in a Box–Behnken experiment with 3 factors (Na-CMC, Tween-80, D-panthenol) and 3 responses (pH, viscosity, release). The selected composition had a desirability of 0.39; expected indicators: release of at least 63.3% (15 min), viscosity ~1,045 mPa·s; thixotropic recovery no worse than: 69% (10 s), 76% (30 s), 79% (60 s; Belenichev et al., 2024b).

Justification of components: Na-CMC—gel former/ mucoadhesive, controlling the “viscosity-release” compromise; Tween-80—solubilizer/release modifier; D-panthenol—moisturizer/potential cytoprotector; benzalkonium chloride—preservative.

(3) Physical, chemical, and rheological characteristics: methods and criteria

pH: If necessary, adjust with buffer to pH 5.5–6.5; justification—physiology of the nasal mucosa and quality guidelines (Patharapankal et al., 2024).

Rheology. Structure recovery ≥70% in 30 s using the 3ITT test.

**Figure 1:**
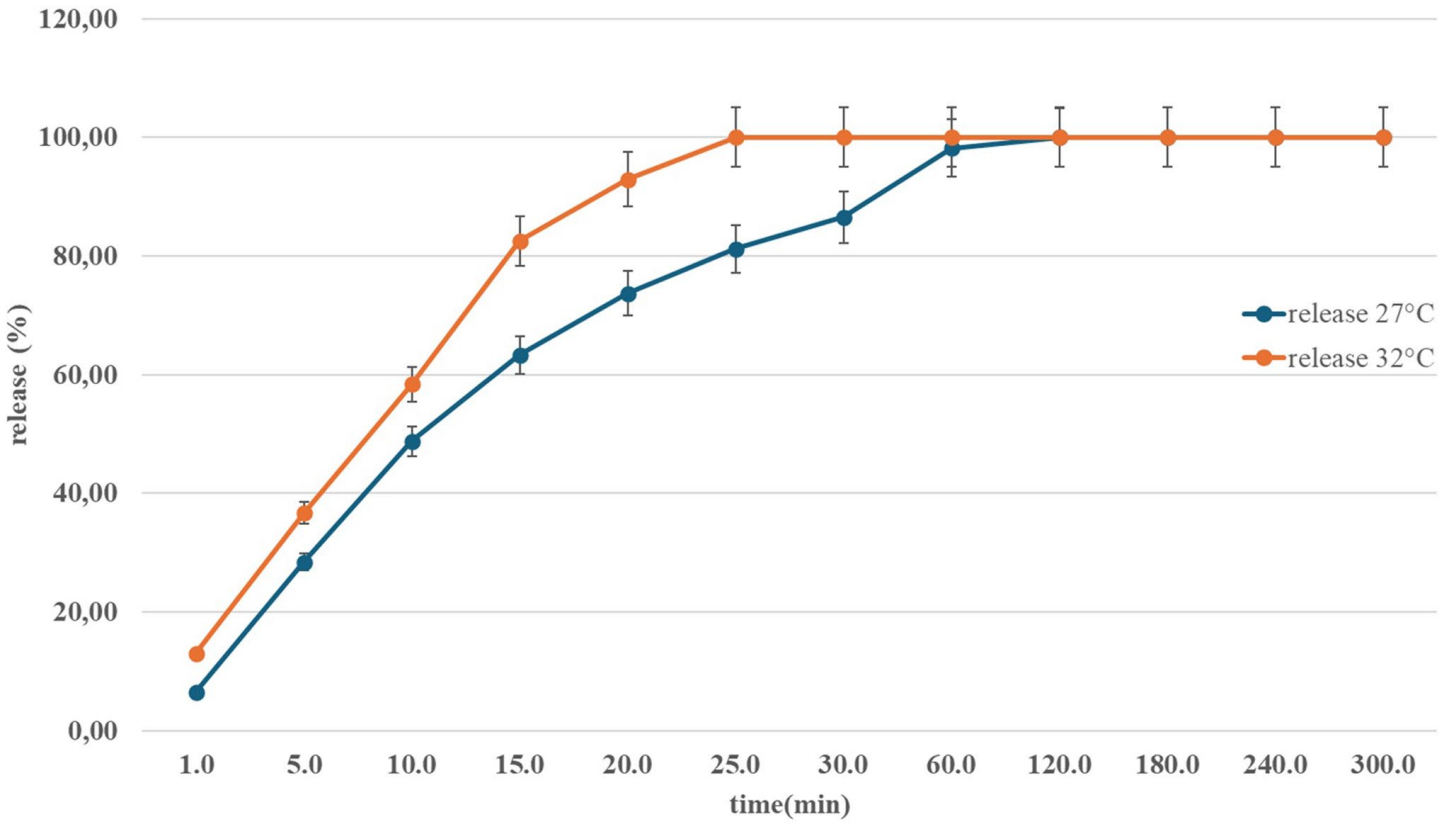
Dynamics of Angiolin release from the nasal form in the temperature range of 27–32 °C.

(4) *In* vitro release of nasal Angiolin using the Franz Cells system at different temperatures (Figure 1).
(5) Preservative

Benzalkonium chloride used at a level of 0.02% (w/w; Belenichev et al., 2024b).

(6) Stability and quality control

Preservation of organoleptic properties, pH, and concentration over a period of 6 months at 8–15 °C has been demonstrated—these data will be further expanded in accordance with ICH protocols (Belenichev et al., 2024b).

### 2.2 Animal characteristics

The experimental research was conducted using a total of 60 white laboratory rats, comprising 50 females and 10 males, each weighing between 220 and 240 grams and approximately 6 months of age. The animals were obtained from the vivarium of the Institute of Pharmacology and Toxicology at the National Medical Academy of Ukraine. Before the experiment began, each animal was examined by a qualified veterinarian to determine its health status. The animals were placed in polycarbonate cages measuring 550x320x180 mm with galvanized steel lids measuring 660x370x140 mm and glass drinking bowls. Five rats were kept in one cage. Each cage was labeled with the study number, species, sex, and animal numbers. The cages were placed on shelves according to the cage numbers indicated on the labels. The following conditions were maintained in the animal room: temperature—20–24 °C, humidity—30–70%, lighting cycle—12 h light/12 h dark. All rats were fed ad libitum with a standard diet for laboratory animals supplied by Phoenix, Ukraine. Water from the municipal water supply (after reverse osmosis and UV sterilization) was given without restriction. Alder sawdust (*Alnus glutinosa*), pretreated by autoclaving, was used as bedding.

All procedures involving animal handling and experimentation adhered to the ethical standards outlined in the “European Convention for the Protection of Vertebrate Animals Used for Experimental and Other Scientific Purposes” (Council of Europe, 1986), as well as relevant national guidelines concerning the ethical use of animals in biomedical research. Ethical approval for the study was granted by the Bioethics Committee of Zaporizhzhia State Medical and Pharmaceutical University (protocol No. 34, dated May 12, 2022).

### 2.3 Experimental model

To create conditions of chronic PH, we used a nitrite-induced method, which leads to significant metabolic and morphologic changes in offspring brain tissue (Wang et al., 2021; Sun and Zhang, 2022). Adult male and female rats were paired at a ratio of 2:4, and pregnancy onset was confirmed by the detection of spermatozoa in vaginal smears. From the 16th to the 21st day of gestation, pregnant females received daily intraperitoneal injections of sodium nitrite solution at a dose of 50 mg/kg to induce moderate PH (Popazova et al., 2023). Control pregnant females received an equivalent volume of physiological saline during the same period.

Each litter contained 8–15 newborn rats. Immediately after birth, the offspring in each litter were randomized, marked, and administered the tested drugs or saline solution starting from day 1 of life. The sex of the pups was not taken into account at this stage, as external sexual dimorphism in newborns is not very pronounced. Determination of sex was performed on postnatal days 7–10; therefore, mortality data, taking into account frequent maternal cannibalism of dead or weakened pups, are presented without separation by sex. Until the 30th day, the pups were kept together with their mother, after which they were placed in separate cages according to the experimental scheme—by type of drug administered and by sex.

Only males were used for subsequent analyses to exclude the possible influence of sex differences on the results. Twenty rats from different litters were randomly selected from each group of males. Each animal was considered an independent experimental unit. The offspring were categorized into the following groups: healthy pups from normoxic pregnancies; control group of PH-exposed pups that received daily physiological saline injections from postnatal days 1 to 30; and two experimental groups of pups after PH receiving daily pharmacological treatments from postnatal days 1 to 30. At the conclusion of the treatment period (postnatal day 30), a subset of animals from each group was euthanized for biochemical, immunohistochemical, and morphometric analyses. The remaining animals were monitored until postnatal day 60 and evaluated using neurophysiological assessment methods. Pharmacological dosing followed established protocols for Piracetam (literature-derived) and Angiolin (determined through preclinical dose-ranging studies, as documented in research reports).

### 2.4 Rationale for the chosen medications and their attributes

The offspring were assigned to four experimental groups, each comprising 20 animals, based on their prenatal conditions and subsequent treatments:

Group 1: Healthy control group consisting of progeny from females that experienced normoxic, uncomplicated pregnancies; these animals received intraperitoneal injections of physiological saline at a dose of 5 μL/g.

Group 2: Prenatal hypoxia (PH) control group, comprising offspring exposed to PH and administered physiological saline intraperitoneally at the same dose (5 μL/g).

Group 3: PH-exposed offspring treated with Angiolin nasal gel administered intranasally at a dosage of 50 mg/kg.

Group 4: PH-exposed offspring treated with Piracetam via intraperitoneal injection at a dose of 500 mg/kg.

Before each drug administration, the animals were weighed on AUX 220 electronic scales (Shimadzu Corporation, Japan). A 10 μL microsyringe was used for intraperitoneal administration, and a micropipette was used for nasal administration. Administration of drugs in the first days after birth was carried out at a temperature of 38–40 °C, maintained using an electric heating pad for premature babies. Before the procedures, the females were temporarily placed in separate cages, where sawdust from their home cage was added; the researcher’s gloves were also wiped with this sawdust to reduce stress levels in the animals. The injection was administered in a separate room to avoid stressing the females and prevent possible litter abandonment. The doses of Angiolin for this age group were calculated experimentally in preclinical studies and presented in reports (Belenichev et al., 2015b).

The pharmacological agents used in this study included the Angiolin nasal gel, formulated according to the method described in section 2.1, and Piracetam (200 mg/mL), sourced from AT “Farmak,” Ukraine.

The blinding method was applied during the stages of testing animal behavior, taking and analyzing biological material. Testing of animals in the open field and two shuttle chambers was carried out alternately with different experimental groups, and biological samples were numbered from 1 and above. The team conducting these studies was blinded. The access team included the study leader.

### 2.5 Anesthesia

In experimental endpoints on days 30 and 60 of the study, the animals were anesthetized using thiopental sodium at a dose of 40 mg/kg. We used Thiopental Sodium lyophilisate for solution for injection in 0.5 g vials (Kyivmedpreparat, Ukraine). Immediately prior to use, an accurately weighed amount of thiopental sodium was dissolved in physiological saline to obtain a 4% solution. The solution was administered to rats intraperitoneally using an insulin syringe at a dose of 0.1 mL of the thiopental sodium solution per 100 grams of body weight. Following anesthesia, blood samples were taken from the celiac artery for further laboratory analysis.

### 2.6 Neurophysiological methods

A subset of the offspring was monitored for 60 days under standard vivarium conditions to evaluate drug effects on developmental milestones, including pelage growth, dental eruption, and eyelid opening. Psychophysiological parameters were assessed on postnatal days 21 and 30. Neurological deficits were quantified using the McGraw Stroke-Index scale in our modification (McGraw, 1977; Belenichev et al., 2024c).

The severity of neurological deficit was determined by summing the scores assigned to specific symptoms. Mild neurological signs (up to 2.5 points) included reduced motor activity, limb weakness, unilateral ptosis, tremors, and circular movements, whereas more severe manifestations (ranging from 3 to 10 points) encompassed limb paresis, hindlimb paralysis, and lateral recumbency.

Exploratory and orientation-related behaviors were assessed using the open-field test. The experimental arena consisted of a large rectangular enclosure (100 × 100 cm) with 40 cm high plastic walls. The floor was constructed from beige-colored plastic marked with a black grid dividing the surface into 25 equal squares (5 × 5). Each rat was placed in a corner of the field, and its behavior was observed for a duration of 3 min, noting horizontal locomotion (number of squares crossed with both forepaws), vertical activity (number of times the animal stood on its hind legs), and exploratory behavior (number of head dips into simulated “burrows”; Gould et al., 2009). The effect of drugs on higher nervous activity was also investigated on the model of one-time training—conditioned passive avoidance reflex (CAPR) without application of amnesic factor (Salmi et al., 1994). The ability of the animal to memorize the applied electric pain stimulus was calculated by the difference of latent time before and after training (after 24 h).

### 2.7 Morphometric methods

At the conclusion of the experimental procedures, animals under anesthesia (is described in subsection 2.5) were euthanized via decapitation using a guillotine. For morphological studies the brains were carefully removed and fixed in Carnoy’s solution for a 24-h period. Subsequently, standard histological procedures were employed, involving paraffin embedding of the tissue and preparation of serial 5-μm frontal sections targeting the CA1 region of the hippocampus. The sections were then deparaffinized using conventional techniques and stained with Einarson’s gallocyanin-chrome alum method to selectively visualize RNA.

Microscopic images of the cerebral cortex were captured using an Axioskop microscope (Zeiss, Germany) and digitized with a COHU-4922 8-bit CCD camera (COHU Inc., United States) connected to the VIDAS-386 image analysis system (Kontron Elektronik, Germany). Morphometric evaluations of neural tissue were conducted in an automated fashion utilizing a macro program developed within the VIDAS-2.5 software environment (Kontron Elektronik, Germany).

The analysis included quantification of the following parameters:

- Neuronal, glial, apoptotic, and structurally altered neuron densities (expressed as the number of cells per mm^2^),
- Cellular composition of the CA1 hippocampal region (expressed as percentages),
- Cross-sectional areas of neuron bodies, glial cells, apoptotic, and damaged neurons (in μm^2^),
- Intracellular RNA content (measured in optical density units, ODU), calculated as the logarithmic ratio of the optical density of the cellular body to that of the surrounding extracellular matrix,
- The index of the ratio of the number of surviving neurons to the total of apoptotic and destructively altered neurons.

### 2.8 Immunohistochemical methods

For immunohistochemical analysis, brain tissues were fixed in Bouin’s solution for 18 h. Following standard histological processing, the samples were embedded in paraffin. Serial sections of the hypothalamus, 14 μm in thickness, were obtained using a rotary microtome and subsequently deparaffinized using conventional techniques.

To investigate the expression of c-Fos and Bcl-2 proteins in the cerebral cortex and hippocampus, the indirect immunofluorescence technique was employed. Tissue sections were incubated with primary antibodies specific to c-Fos and Bcl-2 (Sigma Chemical Co., United States) at 4 °C for 24 h. Following this incubation, the sections were rinsed three times with 0.1 M phosphate buffer. Secondary antibodies—fluorescently labeled goat IgG (Sigma Chemical Co., United States)—were then applied and incubated at room temperature for 60 min. Afterward, the sections were again washed in phosphate buffer.

Immunopositive neurons expressing c-Fos and Bcl-2 were visualized using an Axioskop fluorescence microscope (Zeiss, Germany), and images were captured with a COHU-4922 video camera (COHU Inc., United States). Digital image analysis was performed using the VIDAS-386 system (Kontron Elektronik, Germany). Quality control of immunohistochemical studies was performed using a negative control—samples were processed without the primary antibody, which allowed us to verify the specificity of staining and identify false-positive background signals. The entire automatic image analysis system underwent independent metrological control.

### 2.9 Preparation of biological samples for biochemical analysis

Immediately following extraction, blood was carefully removed from the brain tissue. The cerebral samples were separated from the dura mater and rapidly frozen in liquid nitrogen. The frozen tissue was then pulverized in liquid nitrogen to a fine powder and homogenized at +2 °C in a 10-fold volume of buffer solution containing (in mmol): 250 sucrose, 20 Tris–HCl, and 1 EDTA (pH 7.4).

Isolation of the mitochondrial fraction was performed at +4 °C via differential centrifugation using a Sigma 3-30 k refrigerated centrifuge (Germany). An initial centrifugation at 1000 × g for 7 min was conducted to remove large cellular debris. The resulting supernatant was subsequently centrifuged at 17,000 × g for 20 min. After this step, the supernatant was discarded, and the mitochondrial pellet was retained.

To further purify the mitochondrial fraction, the pellet was resuspended in an extraction buffer supplemented with 0.5 mg/mL bovine serum albumin and centrifuged again at 17,000 × g for 10 min. The final mitochondrial suspension was prepared in the same extraction medium and contained 40–60 mg of protein per mL (Belenichev et al., 2024c). The quality control of neuronal mitochondria isolation was performed using electron microscopy. For this purpose, the mitochondria suspension was incubated for 5 min at room temperature, then fixed with glutaraldehyde, and subsequently with osmic acid. After that, it was washed several times with 50% ethanol. It was then treated with a 1% solution of osmium tetroxide in buffer for 1.5 h and dehydrated in solutions of alcohols of increasing concentration (70% alcohol was saturated with uranyl acetate) and poured into Epon-812 epoxy resin diluted with acetone.

### 2.10 Biochemical methods

The activity of succinate dehydrogenase (SDH) in mitochondrial lysates was quantified using the Succinate Dehydrogenase Activity Assay Kit (Colorimetric; Cat. No. ab228560, Abcam, United Kingdom), following the manufacturer’s protocol. Optical density was measured at 600 nm. Similarly, ATP levels in the mitochondrial lysates were assessed using the ATP Assay Kit (Colorimetric; Cat. No. ab83355, Abcam, UK), with Optical density recorded at 570 nm.

The concentrations of lactate, malate, and isocitrate were quantified using a spectrophotometric method. The malate assay is based on the enzymatic conversion of malate to oxaloacetate via malate dehydrogenase, accompanied by the reduction of NAD to NADH. This reaction results in an increase in optical density at 340 nm, which is directly proportional to the malate concentration.

The method of isocitrate concentration determination is based on its enzymatic conversion to *α*-ketoglutarate by isocitrate dehydrogenase, accompanied by the simultaneous reduction of NADP to NADPH. This reaction is associated with an increase in optical density at 340 nm, which is equimolar to the amount of isocitrate present.

The determination of lactate concentration is based on its enzymatic conversion to pyruvate in the presence of lactate dehydrogenase, accompanied by the simultaneous reduction of NAD to NADH. This reaction results in an increase in optical density at 340 nm, which is equimolar to the amount of lactate in the sample.

All analytical procedures were conducted using certified reagents (Sigma Chemical Co., United States) with optical density measurements performed on an Eppendorf BioSpectrometer (Eppendorf, United States).

### 2.11 Immunoenzymatic methods

Nitrotyrosine levels in the cytosolic fraction of brain homogenates were measured using a solid-phase sandwich enzyme-linked immunosorbent assay (ELISA), employing the ELISA Kit (Catalog No. HK 501–02, Hycult Biotech, Uden, Netherlands) in accordance with the manufacturer’s protocol.

The activity of inducible nitric oxide synthase (iNOS) in the cytosolic brain fraction was measured using the ELISA Kit (MyBioSource, San Diego, CA, United States; Catalog No. MBS023874), in accordance with the supplied instructions.

Cytosolic Cu/Zn-superoxide dismutase (SOD1) activity was evaluated using the Rat SOD1/Cu-Zn SOD Sandwich ELISA Kit (Catalog No. LS-F4234, LSbio, United States), following the recommended protocol.

Glutathione peroxidase 4 (GPX4) activity in blood serum was determined by enzyme immunoassay utilizing the GPX-4 ELISA Kit specific for rat phospholipid hydroperoxide glutathione peroxidase (Catalog No. MBS934198, MyBioSource, United States), according to the manufacturer’s guidelines.

The level of glutathione peroxidase 1 (GPX1) in the cytosolic fraction of brain homogenates was measured using a solid-phase sandwich enzyme-linked immunosorbent assay (ELISA) with the Rat Glutathione Peroxidase 1 ELISA Kit (Catalog No. MBS3809062, MyBioSource, Inc., United States), following the manufacturer’s protocol.

GABA transaminase activity was assessed in the same cytosolic fraction using the Rat 4-aminobutyrate aminotransferase, mitochondrial (ABAT) ELISA Kit (Catalog No. MBS284363, MyBioSource, Inc., United States).

Glutamate decarboxylase (GAD) concentrations were measured using the Rat GAD ELISA Kit (Catalog No. MBS263620, MyBioSource, United States) according to the manufacturer’s instructions.

Gamma-aminobutyric acid (GABA) levels were determined using the Rat Gamma-Aminobutyric Acid ELISA Kit (Catalog No. MBS269152, MyBioSource, Inc., United States) following standard assay protocols.

NADH-malate dehydrogenase (MDH2) activity in the mitochondrial fraction of brain homogenates was quantified using the Rat Malate Dehydrogenase, Mitochondrial ELISA Kit (Catalog No. MBS9712304, MyBioSource, Inc., United States), following the specified protocol.

Creatine phosphokinase B-type (CKB-B) activity in mitochondrial lysates was measured using the Rat CPK ELISA Kit (Catalog No. abx258101, Abbexa Ltd., United Kingdom), following the recommended procedure.

All measurements were performed using a microplate ELISA-Reader Sirio S (SEAC, Italy).

### 2.12 Statistical analysis

All experimental data were processed using StatisticaR for Windows 6.0 (StatSoft Inc., license No. AXXR712D833214FAN5), SPSS version 16.0, and Microsoft Office Excel 2010. Data normality was checked using the Shapiro–Wilk test. Student’s t-test was used for paired comparisons of normally distributed data. For multiple comparisons of means between groups with prenatal hypoxia, angiolin treatment, and piracetam treatment, one-way analysis of variance (ANOVA) was used. Pearson’s correlation coefficient (r) was used to analyze the relationship between parameters.

The sample size was calculated for the survival rate of rats in groups using the formula:


$$n={\left(Z_{\propto }+Z_{\beta }\right)}^{2}\frac{p_{1}(1-p_{1})+p_{2}(1-p_{2})}{\Delta^{2}},$$

where *Δ* (effect size) was the minimum clinical difference between the proportions equal to 50%, the significance level *α* was set at 0.05, and the power of the criterion was −0.80 (*β* = 0.2).


$$n={\left(1.645+0.842\right)}^{2}\frac{0.338(1-0.338)+0.806(1-0.806)}{0.5^{2}}=9.368.$$

Thus, the sample size was 10.

To assess the magnitude of the effect for continuous data, Cohen’s d criterion ($d=\frac{\bar{x}-\bar{y}}{s_{\text{pooled}}}$), was used, and for proportions, Cohen’s h criterion was used $\;h=2(\arcsin(\sqrt{p_{1}})-$ $\arcsin(\sqrt{p_{2}}))$.

The decision on the size of the effect was made on a scale:

d(h) = 0.2: Small effect size.

d(h) = 0.5: Medium effect size, noticeable to an attentive observer.

d(h) ≥ 0.8: Large effect size.

To verify the validity, reliability of assessments, and internal consistency of the items on the behavioral scales, Cronbach’s alpha was calculated (α):


α=N∗r/(1+r(N−1)),

where r is the average linear correlation coefficient.

95% Confidence intervals were calculated using the formulas:

- for continuous data—$[95\%\mathrm{CI}]=\bar{X}\pm Z_{\propto /2}\frac{\sigma }{\sqrt{n}}$;
- for proportions—$[95\%\mathrm{CI}]=p\pm Z_{\propto }\sqrt{p(1-p)}$

For all types of analysis, differences were considered statistically significant at *p* < 0.05.

## 3 Results

Our biochemical studies of the brain of 1-month-old rats after PH revealed persistent disorders of energy metabolism (Table 2). We found that PH leads to the development of mitochondrial dysfunction in the brain of the offspring. Thus, we registered in the brain of 1-month-old rats of the control group a decrease in creatine phosphokinase (CKB-B-type) expression (*p* < 0.05), and mitochondrial malate dehydrogenase (mtMDH; *p* < 0.05), which indicated a violation of the main functions of mitochondria—energy production and transport. Additionally, there was a significant decrease in malate levels (*p* < 0.05) and inhibition of mitochondrial succinate dehydrogenase (SDH) activity (*p* < 0.05) compared to the intact group, indicating discoordination in the Krebs cycle.

**Table 2:** Effect of Angiolin Gel and Piracetam on some markers of brain energy metabolism of 1-month-old rats after PH (Mean ± SEM).

Groups of animals	Isocitrate, μmol/g	Lactate, μmol/g	Malate, μmol/g	SDH activity, mU\ml	MDH2, ng\ml	АТФ, μmol/g	CKB-B, pg./ml
Intact (Rats born from rats with normal pregnancies) *(n = 10)*	0.68 ± 0.01	2.17 ± 0.01	0.44 ± 0.02	7.84 ± 0.10	7.4 ± 0.10	3.55 ± 0.02	473.5 ± 9.2
PH (Сontrol) (Rats after PH) *(n = 10)*	0.42 ± 0.01^1^	4.77 ± 0.11^1^	0.30 ± 0.02^1^	4.73 ± 0.15^1^	4.7 ± 0.11^1^	2.88 ± 0.01^1^	315.5 ± 11.0^1^
PH + Angiolin gel, 50 mg/kg *(n = 10)*	0.65± 0.02*	2.75 ± 0.21*#	0.52 ± 0.02*#	6.72 ± 0.12*	8.2 ± 0.10*#^1^	3.44 ± 0.01*	431.4 ± 12.3*^#^
PH + Piracetam, 500 mg/kg *(n = 10)*	0.51 ± 0.01*^1^	5.32 ± 0.10*^1^	0.35 ± 0.01*^1^	5.91 ± 0.10*^1^	5.0 ± 0.20^1^	2.98 ± 0.01*^1^	392.1 ± 10.2*^1^

*p < 0.05 in relation to the indicators of the control group; ^1^*p* < 0.05 in relation to the indicators of the intact group; #*p* < 0.05 in relation to the indicators of the group of animals after PH receiving Piracetam.

In the brain of 30-day-old rats of the control group, a change in the GABAergic system was observed (Table 3), as evidenced by increased expression of GABA-T (*p* < 0.05), and GAD (*p* < 0.05), and decreased level of GABA (*p* < 0.05) in the cytosol of the brain homogenate compared with similar parameters of the intact group, which possibly showed activation of the Roberts shunt. In addition, activation of glycolysis—increase of lactate level in the cytosol of brain homogenate was observed (*p* < 0.05). However, activation of compensatory energetic reactions in conditions of mitochondrial dysfunction does not provide a sufficient level of energy, as evidenced by the low level of ATP (*p* < 0.05) in brain mitochondria of 30-month-old animals of the control group compared to the intact group. Administration of Angiolin gel (50 mg/kg) intranasally to animals after PH led to a decrease in the effects of mitochondrial dysfunction in the brain of 30-day-old rats (Table 2). Thus, it was revealed an increase in ATP concentration in mitochondria due to normalization of oxidative reactions of Krebs cycle—increase of malate (*p* < 0.05), and isocitrate (*p* < 0.05) levels in cytosol, increase of SDH activity (*p* < 0.05), mitochondrial MDH (*p* < 0.05) and m-CPK (*p* < 0.05) in brain mitochondria of 30-day-old animals after PH compared to control group. Furthermore, Angiolin administration led to a normalization of GABAergic system parameters. A significant increase in GABA levels (*p* < 0.05) was observed, accompanied by a decrease in the expression of both GABA-T (*p* < 0.05) and GAD (*p* < 0.05) in the brain cytosol of 30-day-old rats after PH compared to control group. These values did not significantly differ from similar parameters of the intact group (Table 3). Course nasal administration of Angiolin to offspring after PH provided and decrease of lactate concentration in brain cytosol (*p* < 0.05) in comparison with the control group, which indicates the limitation of low-productive glycolysis and possible risks of lactate acidosis. In contrast, a 30-day course of Piracetam administration to offspring following PH did not demonstrate a comparable level of efficacy to Angiolin (*p* < 0.05) in mitigating mitochondrial dysfunction or energy deficiency. Moreover, Piracetam significantly increased lactate levels (*p* < 0.05) in the brains of 30-day-old rats, which may have an adverse effect on brain function. A one-way analysis of variance was performed to verify the statistical significance of the difference in mean values between the intact, control and treatment (with angiolin and piracetam) groups. The calculated F-statistic of 5.96 is higher than the critical *F*-value for a significance level of 0.05, indicating statistically significant differences between the groups. Thus, it can be argued that the type of treatment has a statistical effect on the dependent variable. To clarify the differences between the groups, a paired Student’s t-test was used.

**Table 3:** Effect of Angiolin gel and Piracetam on some markers of the GABAergic system in the brain of one-month-old rats after PH (Mean ± SEM).

Groups of animals	GABA ng\ml	GAD ng\ml	GABA-T ng\ml
Intact (Rats born from rats with normal pregnancies) *(n = 10)*	94.7 ± 7.1	4.5 ± 0.2	4.7 ± 0.2
PH (Сontrol) (Rats after PH) *(n = 10)*	52.1 ± 4.5^1^	7.8 ± 0.4^1^	7.1 ± 0.5^1^
PH + Angiolin gel, 50 mg/kg *(n = 10)*	85.1 ± 4.2*#	4.8 ± 0.2*#	5.4 ± 0.2*#
PH + Piracetam, 500 mg/kg *(n = 10)*	58.2 ± 5.6^1^	7.3 ± 0.4^1^	7.0 ± 0.3^1^

*p < 0.05 in relation to the indicators of the control group; ^1^*p* < 0.05 in relation to the indicators of the intact group; #*p* < 0.05 in relation to the indicators of the group of animals after PH receiving Piracetam.

Our study demonstrated that PH induced activation of inducible nitric oxide synthase (iNOS; *p* < 0.05) in the cytosolic fraction of the brain, leading to excessive nitric oxide (NO) production in 30-day-old rat pups. This hyperproduction contributed to the development of nitrosative stress, as evidenced by a significant increase in nitrotyrosine levels (*p* < 0.05; Table 4). In the brains of one-month-old offspring subjected to PH, the progression of nitrosative stress occurred against a background of pronounced antioxidant deficiency. Specifically, we observed a significant decrease in the expression of Cu/Zn-superoxide dismutase (Cu/Zn-SOD; *p* < 0.05), glutathione peroxidase 1 (GPX1; *p* < 0.05), and glutathione peroxidase 4 (GPX4; *p* < 0.05) compared to the intact control group. Intranasal administration of Angiolin immediately after birth in animals after PH significantly reduced nitrotyrosine levels (*p* < 0.05) and iNOS expression (*p* < 0.05), while increasing the expression of Cu/Zn-SOD (*p* < 0.05), GPX1 (*p* < 0.05), and GPX4 (*p* < 0.05) in the cytosolic brain fraction of 30-day-old rat pups. In contrast, treatment with Piracetam at a dose of 500 mg/kg had no significant effect on the parameters of the antioxidant defense system or on indicators of nitrosative stress in the brains of 30-day-old rat pups after PH (Table 5).

**Table 4:** Effect of Angiolin gel and Piracetam on the content of nitrotyrosine and iNOS in the cytosol of brain homogenate of 1-month-old rats after PH (Mean ± SEM).

Groups of animals	Nitrotyrosine ng/ml	iNOS, ng/ml
Intact (Rats born from rats with normal pregnancies) *(n = 10)*	3.0 ± 0.11	3.77 ± 0.22
PH (Сontrol) (Rats after PH) *(n = 10)*	18.7 ± 1.27^1^	15.2 ± 1.33^1^
PH + Angiolin gel, 50 mg/kg *(n = 10)*	5.8 ± 0.4*#^1^	4.17 ± 0.21*#
PH + Piracetam, 500 mg/kg *(n = 10)*	11.2 ± 1.2^1*^	12.4 ± 1.82^1^

*p < 0.05 in relation to the indicators of the control group; ^1^*p* < 0.05 in relation to the indicators of the intact group; #*p* < 0.05 in relation to the indicators of the group of animals after PH receiving Piracetam.

**Table 5:** Effect of Angiolin gel and Piracetam on some indices of antioxidant system of brain of one-month-old rats after PH (Mean ± SEM).

Groups of animals	Cu/ZnSOD, pg./ml	GPX1, pg./ml	GPX4, pg./ml
Intact (Rats born from rats with normal) pregnancies *(n = 10)*	88.4 ± 3.7	33.2 ± 1.7	66.4 ± 4.8
PH (Сontrol) (Rats after PH) *(n = 10)*	51.6 ± 2.2^1^	19.4 ± 0.9^1^	47.7 ± 2.1^1^
PH + Angiolin gel, 50 mg/kg *(n = 10)*	77.5 ± 2.8^*#1^	38.4 ± 1.5^*#1^	63.6 ± 3.1^*#^
PH + Piracetam, 500 mg/kg *(n = 10)*	53.6 ± 2.8^1^	20.2 ± 2.3	48.0 ± 2.2^1^

*p < 0.05 in relation to the indicators of the control group; ^1^*p* < 0.05 in relation to the indicators of the intact group; #*p* < 0.05 in relation to the indicators of the group of animals after PH receiving Piracetam.

**Figure 2:**
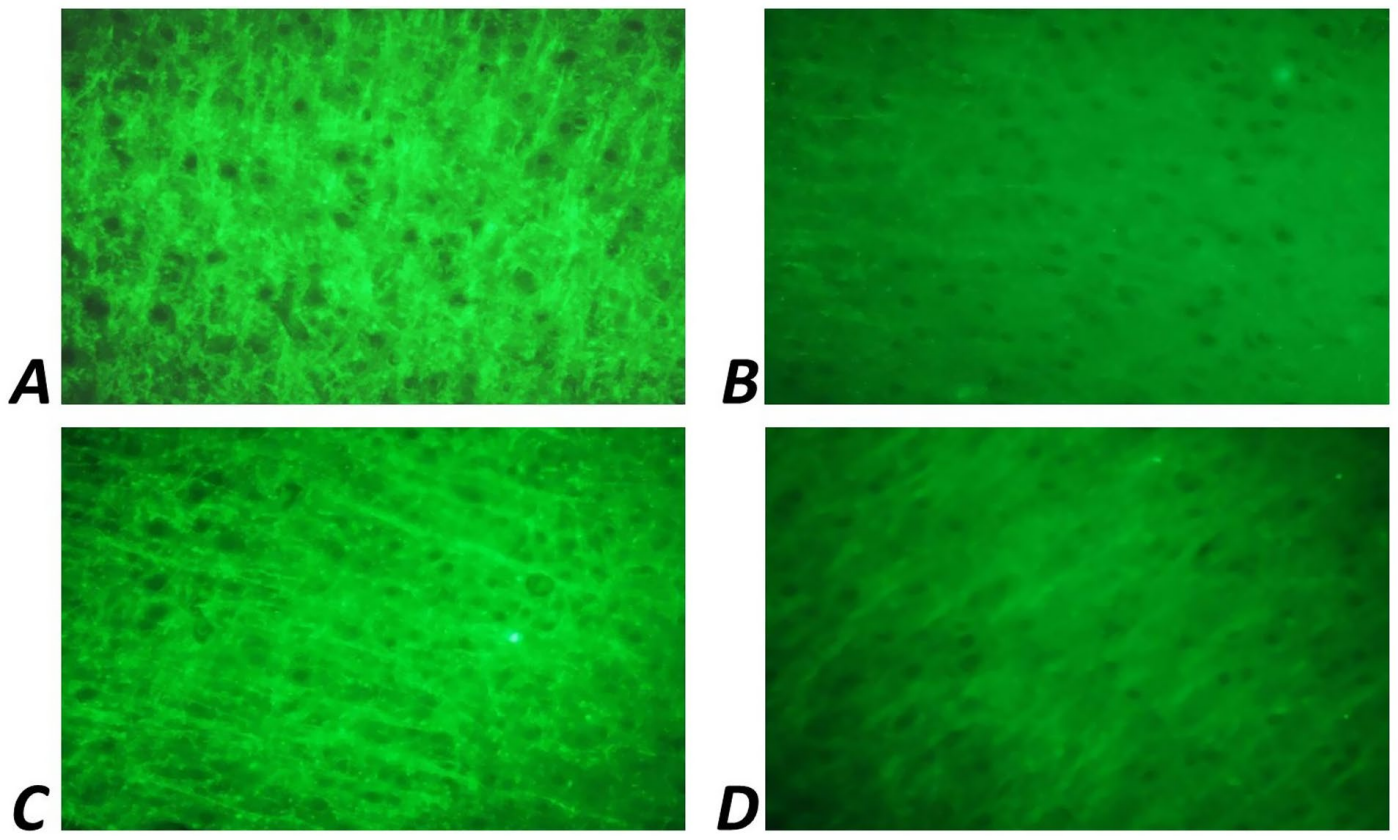
Fluorescence of Fos-positive neurons of CA1 zone of hippocampus of 1-month-old rats. Ob. x 100: **(A)** intact group; **(B)** rats after PH (control); **(C)** rats after PH, which received a 30-day course of Angiolin gel; **(D)** rats after PH, which received a 30-day course of Piracetam.

Immunohistochemical analysis revealed that in 30-day-old rat pups subjected to prenatal hypoxia (PH), the number of Fos-positive neurons in the CA1 region of the hippocampus was significantly reduced compared to the intact control group. Intranasal administration of Angiolin immediately after birth resulted in a significant increase in the number of Fos-positive neurons in the CA1 hippocampal area of 30-day-old pups compared to the PH control group (Table 6; Figure 2). Furthermore, it was shown that intranasal Angiolin administration normalized the expression levels of the anti-apoptotic protein Bcl-2 in hippocampal neurons. Bcl-2 is considered a metal-containing protein that has properties of neutralizing free radicals and inhibiting the development of apoptosis. (Table 6).

**Table 6:** Effect of Angiolin gel and Piracetam on the expression of early response genes (c-fos) by the number of Fos-positive neurons and Bcl-2 content in neurons of hippocampus CA1 of one-month-old rats after PH (Mean ± SEM).

Groups of animals	Number of Fos-positive neurons in 1 mm^2^	Number of Bcl-2-positive neurons in 1 mm^2^
Intact (Rats born from rats with normal pregnancies) *(n = 10)*	72.3 ± 2	201.1 ± 6
PH (Сontrol) (Rats after PH) *(n = 10)*	38.0 ± 1.4^1^	144.2 ± 7^1^
PH + Angiolin gel, 50 mg/kg *(n = 10)*	68.7 ± 1.3^*#1^	190.5 ± 10^*#^
PH + Piracetam, 500 mg/kg *(n = 10)*	40.1 ± 1.7^1^	145.8 ± 5^1^

*p < 0.05 in relation to the indicators of the control group; ^1^*p* < 0.05 in relation to the indicators of the intact group; #*p* < 0.05 in relation to the indicators of the group of animals after PH receiving Piracetam.

Morphometric study of the brain of 30-day-old rats after PH showed a significant decrease in the density of hippocampal CA1 neurons (*p* < 0.05) compared to the intact group (Table 7). At the same time, there was a decrease in the area of neuron bodies (*p* < 0.05) with decreased RNA content in them (*p* < 0.05) compared to healthy animals (intact group). PH also exerted a negative effect on glial cell density in the hippocampus (Table 8), as evidenced by a significant decrease in glial cell count (*p* < 0.05), reduction in glial cell body area (*p* < 0.05), and lower RNA content within these cells (*p* < 0.05), when compared to intact animals. Furthermore, in the CA1 region of the hippocampus, an increase in neuroapoptosis was observed, indicated by a higher number of neurons exhibiting apoptotic features (*p* < 0.05) in the group of rats after PH. Administration of Angiolin gel intranasally to animals after PH demonstrated a neuroprotective effect, which was expressed in the increase in the density (*p* < 0.05) and body area (*p* < 0.05) of neurons, as well as an elevation in RNA content (*p* < 0.05), suggesting enhanced morphofunctional activity of hippocampal neurons. Angiolin gel also exhibited a neuroprotective effect on glial elements, promoting an increase in glial cell density and restoring RNA content (*p* < 0.05) to levels comparable to those of healthy animals. These findings are consistent with the results of immunohistochemical analysis, which demonstrated the positive impact of Angiolin gel on the transcriptional activity of hippocampal neurons in neonatal rats. Administration of Angiolin gel immediately after birth for 30 days significantly reduced the number of apoptotic and degeneratively altered neurons in the hippocampus of 30-day-old rats (*p* < 0.05) in comparison with similar parameters of the group of untreated animals after PH (control). In contrast, Piracetam administered under the same protocol failed to produce a comparable neuroprotective effect (Tables 7, 8).

**Table 7:** Morpho-functional characteristics of hippocampal CA1 neurons of rat after PH on 30 days of life (Mean ± SEM).

Groups of animals	Density of neurons, cells/mm^2^	Area of neuron bodies, μm^2^	RNA content in neurons, U_OD_	Density of cells with signs of apotosis per 1 mm^2^
Intact (Rats born from rats with normal pregnancies) (*n* = 10)	1,427 ± 11	62.4 ± 0.9	10.78 ± 0.11	5.07 ± 0.20
PH (Rats with prenatal hypoxia) (control) (*n* = 10)	1,272 ± 7^1^	57.1 ± 0.7^1^	9.00 ± 0.03^1^	24.2 ± 0.77^1^
PH + Angiolin gel, 50 mg/kg (*n* = 10)	1,355 ± 5*^1^	61.1 ± 0.5*	12.83 ± 0.08^*#1^	12.7 ± 0.35^*#1^
PH + Piracetam, 500 mg/kg (*n* = 10)	1,298 ± 9*^1^	58.4 ± 0.9^1^	9.97 ± 0.05*^1^	19.2 ± 0.12*^1^

*p < 0.05 in relation to the indicators of the control group; ^1^*p* < 0.05 in relation to the indicators of the intact group; #*p* < 0.05 in relation to the indicators of the group of animals after PH receiving Piracetam.

**Table 8:** Morpho-functional characteristics of hippocampal CA1 glial cells of rat after PH on 30 days of life (Mean ± SEM).

Groups of animals	Density of glial cells, cells/mm^2^	Area of glial cell bodies, μm^2^	RNA content in glial cells, U_OD_
Intact (Rats born from rats with normal pregnancies) *(n = 10)*	438 ± 7	22.8 ± 0.08	3.32 ± 0.02
PH (Сontrol) (Rats after PH) *(n = 10)*	400 ± 5^1^	20.3 ± 0.10^1^	2.52 ± 0.02^1^
PH + Angiolin gel, 50 mg/kg *(n = 10)*	437 ± 8*	23.4 ± 0.07*#	3.32 ± 0.02*#
PH + Piracetam, 500 mg/kg *(n = 10)*	425 ± 10	20.7 ± 0.05^1^	2.85 ± 0.02*^1^

*p < 0.05 in relation to the indicators of the control group; ^1^*p* < 0.05 in relation to the indicators of the intact group; #*p* < 0.05 in relation to the indicators of the group of animals after PH receiving Piracetam.

PH modeling resulted in PH modeling resulted in impaired physical and cognitive development in the offspring (Tables 9–12), accompanied by the emergence of neurological deficits of moderate severity, as assessed by the McGraw scale. PH led to a sharp increase in early postnatal mortality. The rats that underwent PH had obvious signs of cognitive-mnesic disorders, which was evidenced by inhibition of orientation-research activity [decrease in the number of horizontal (*p* < 0.05), vertical movements (*p* < 0.05), the number of peeks in the “burrow” (*p* < 0.05)], as well as a decrease in the latent period of CAPR (*p* < 0.05) compared to similar parameters of the intact group. Administration of Angiolin gel immediately after birth for 30 days to rats after PH resulted in the birth of healthier offspring, which did not differ from intact animals in terms of coat coverage, teething, and eye opening. Nasal administration of Angiolin significantly reduced by 47% the early postnatal mortality of offspring (*p* < 0.05) that had undergone PH. Administration of Angiolin gel almost completely eliminated the manifestation of neurological deficit (motor disorders, impaired coordination, as well as tactile and pain sensitivity) in 60-day-old rats. An important point in the effect of Angiolin gel was a positive influence on such parameters of cognitive-mnestic functions of the CNS of 60-day-old rats after PH, as preservation of the memory trace (increase in the latent period of CAPR; *p* < 0.05) and activation of the orientation-research activity of animals [increase in the number of horizontal (*p* < 0.05) and vertical movements (*p* < 0.05), the number of “peeks into the “burrow” (*p* < 0.05)] in comparison with the parameters of animals of the control group. The use of Piracetam had significantly less therapeutic effect (*p* < 0.05). This effect of Angiolin gel indicated its significant neuroprotective effect and is an experimental justification for clinical application in neonatological, neurological and pediatric practice for neuroprotection of prenatal CNS lesions. Cronbach’s alpha (*α*) was calculated to verify the reliability of the assessments and the internal consistency of the items on the behavioral scales. In all groups, Cronbach’s alpha exceeded 0.7 (0.7 < α < 0.9), indicating acceptable and good consistency of the scale.

**Table 9:** Effect of Angiolin gel and Piracetam on physical development of newborn rats after PH (Mean ± SEM).

Groups of animals	Hair covering (days)	Teeth eruption (days)	Opening of eyes (days)
Intact (Rats born from rats with normal pregnancies) *(n = 10)*	11.0 ± 0.471	11.1 ± 0.316	13.4 ± 0.516
PH (Сontrol) (Rats after PH) *(n = 10)*	14.2 ± 0.422^1^	12.9 ± 0.316	16.8 ± 0.422^1^
PH + Angiolin gel, 50 mg/kg *(n = 10)*	11.7 ± 0.483*	11.0 ± 0.471*	13.2 ± 0.422*
PH + Piracetam, 500 mg/kg *(n = 10)*	13.5 ± 0.527^1^	11.8 ± 0.422	15.7 ± 0.483^1^

*p < 0.05 in relation to the indicators of the control group; ^1^*p* < 0.05 in relation to the indicators of the intact group; #*p* < 0.05 in relation to the indicators of the group of animals after PH receiving Piracetam.

**Table 10:** Effect of Angiolin gel and Piracetam on survival and development of neurological disorders in offspring after PH at 30 days of life (Mean ± SEM [Min; Max]).

Groups of animals	Average score on the McGraw scale	Birth/survivor ratio	% of survivors
Intact (Rats born from rats with normal pregnancies) *(n = 10)*	0.00 ± 0.00	(72)65	90.0 [71;100]
PH (Сontrol) (Rats after PH) *(n = 10)*	4.9 ± 0.7^1^ [4.7;5.1]	(121) 41	33.8^1^ [4.8;62.8]
PH + Angiolin gel, 50 mg/kg *(n = 10)*	1.0 ± 0.5^*#1^ [0.7;1.3]	(116) 98	80.6^*#^ [56.1;100]
PH + Piracetam, 500 mg/kg *(n = 10)*	3.4 ± 0.8^1^ [3.1;3.7]	(123) 60	48.7^1^* [17.8;79.6]

*p < 0.05 in relation to the indicators of the control group; ^1^*p* < 0.05 in relation to the indicators of the intact group; #*p* < 0.05 in relation to the indicators of the group of animals after PH receiving Piracetam.

In round brackets—number of born animals, after brackets—survived by 30 days. The effect of Angiolin on the development of neurological disorders in offspring after PH was assessed using Cohen’s d and compared with the control group: $\;d=\frac{4.9-1.0}{\sqrt{\frac{0.31^{2}+0.47^{2}}{2}}}=6.8$ and compared to the group that treated with Piracetam: $d=\frac{3.4-1.0}{\sqrt{\frac{0.51^{2}+0.47^{2}}{2}}}=4.8.$ Such Cohen’s d values indicate a large effect. To assess the effect of Angiolin on the survival rate, Cohen’s h was calculated for the control group: $h=2(\arcsin(\sqrt{0.81})-\arcsin(\sqrt{0.34})=0.6)$. To assess the effect of Angiolin on survival compared to Piracetam, Cohen’s h was calculated as follows: $h=2(\arcsin(\sqrt{0.81})-\arcsin(\sqrt{0.49})=0.4)$. These values correspond to the average effect.

**Table 11:** Effect of Angiolin gel and Piracetam on orientation-research activity in offspring after PH at 60 days of life (Mean ± SEM).

Groups of animals	Number of horizontal movements	Number of standing on hind legs	Number of peeking through the hole in the floor
Intact (Rats born from rats with normal pregnancies) *(n = 10)*	47.2 ± 3.5	17.7 ± 1.5	34.2 ± 3.2
PH (Сontrol) (Rats after PH) *(n = 10)*	22.7 ± 3.7^1^	9.0 ± 1.5^1^	15.4 ± 2.3^1^
PH + Angiolin gel, 50 mg/kg *(n = 10)*	40.7 ± 3.1*	15.2 ± 1.2*#	28.5 ± 1.5*#
PH + Piracetam, 500 mg/kg *(n = 10)*	37.8 ± 2.3*	12.5 ± 3.0	19.7 ± 1.2*

*p < 0.05 in relation to the indicators of the control group; ^1^*p* < 0.05 in relation to the indicators of the intact group; #*p* < 0.05 in relation to the indicators of the group of animals after PH receiving Piracetam.

**Table 12:** Effect of Angiolin gel and Piracetam on latent time of conditioned passive avoidance reflex (CAPR) in offspring after PH at 60 days of life (Mean ± SEM).

Groups of animals	Latency time before training, sec	Latency time after training, sec
Intact (Rats born from rats with normal pregnancies) *(n = 10)*	12.7 ± 2.2	129.8 ± 10.0
PH (Сontrol) (Rats after PH) *(n = 10)*	17.0 ± 1.5	57.0 ± 2.3^1^
PH + Angiolin gel, 50 mg/kg *(n = 10)*	14.0 ± 2.4	97.4 ± 7.0*#
PH + Piracetam, 500 mg/kg *(n = 10)*	12.6 ± 2.3	63.5 ± 4.3*

*p < 0.05 in relation to the indicators of the control group; ^1^*p* < 0.05 in relation to the indicators of the intact group; #*p* < 0.05 in relation to the indicators of the group of animals after PH receiving Piracetam.

The course administration of Piracetam to the offspring after PH led to a decrease in early postnatal lethality (*p* < 0.05), preservation of the memory trace after training in a two-shuttle chamber (*p* < 0.05) and increased the number of peeks into the hole (*p* < 0.05) in the open-field test. However, the registered improvement of CNS parameters in the group of animals after PH, receiving Piracetam was less significant (*p* < 0.05) than in the similar group, receiving Angiolin gel as a treatment. Piracetam did not affect other indicators of offspring development and CNS functions.

## 4 Discussion

The model of PH by intraperitoneal administration of sodium nitrite (50 mg/kg) to pregnant female rats from day 16 to 21 of gestation, which we used in the experiment, leads to hemic hypoxia due to methemoglobin formation, which is combined with tissue hypoxia due to dissociation of oxidation and phosphorylation processes rather than due to direct toxic effect of sodium nitrite. Disruption of blood oxygen transport function in pregnant female rats leads to impaired uteroplacental circulation and oxygen starvation of the fetus or embryo (Office of Environmental Health Hazard Assessment (OEHHA), 2000). Administration of sodium nitrite at a dose of 50 mg/kg leads to hypoxia of medium severity in adult individuals, according to the criteria proposed by Ivanitskaya (1976). This approach to PH modeling leads to the development of postnatal CNS disorder. Such a model of PH provides a sufficiently large opportunity not only to objectively study the general physical and psycho-emotional development of the offspring, higher CNS functions, but also allows us to evaluate the effectiveness of experimental neuroprotective therapy after PH (Thompson et al., 2015; Piešová et al., 2020; Indriawati et al., 2018; Xue et al., 2020). Our previous studies have shown that modeling of PH with sodium nitrite leads to persistent CNS disorders—significant early postnatal lethality, persistent impairment of motor activity and inhibition of cognitive-mnestic functions of the CNS in offspring against the background of decreased expression of endogenous cyto- and neuroprotective factors (HSP70, HIF-1; Aliyeva et al., 2025; Belenichev et al., 2024a).

We also found a significant disturbance of myocardial nitroxidergic system in rats after PH, particularly the disturbance of eNOS/iNOS expression ratio on the background of NO deficiency and nitrotyrosine increase, which suggests the activation of nitrosative stress in the brain of offspring after PH (Belenichev et al., 2024d). Nitrosative stress leads to a decrease in the function of proteins in the chain of electron carriers, ATP-ase activity, selective action of transport proteins and ion channels and, ultimately, lead to impaired secretory, incretin, and transport functions of neurons, and, as a consequence, to the development of cognitive deficits (Butterfield and Boyd-Kimball, 2020; Orfali et al., 2024; Ebanks and Chakrabarti, 2022). The role of NO derivatives in suppressing gene activity and reducing the level of various transcription factors has been demonstrated (de la Cruz-Ojeda et al., 2021; Andrabi et al., 2023). Cytotoxic derivatives of NO (peroxynitrite and nitrosonium ion) participate in the mechanisms of formation of mitochondrial dysfunction and increase it under hypoxia (Zong et al., 2024; Chen et al., 2024; Zhou et al., 2024).

Mitochondrial dysfunction due to PH is the main cause of persistent energy deficit and inhibition of reactions of ATP formation, which we found in this study. The changes in the state of the GABAergic system in the brain of offspring after PH are likely to occur as a compensatory activation of an additional shunt of energy formation under conditions of Krebs cycle inhibition (Riljak et al., 2016). Thus, inhibition of a-ketoglutarate oxidation leads to the activation of neuronal GAD and conversion of glutamate into GABA, and then, upon activation of GABA-T, into succinic semialdehyde, which, turning into succinate, is oxidized in the Krebs cycle (Andersen and Schousboe, 2023; Andersen, 2025). However, the inhibition of the Krebs cycle at the isocitrate-succinate site and SDH suppression, which we have found, indicates inhibition of the succinatoxidase pathway of proton delivery to the respiratory chain and inability to use additionally formed succinate in the Roberts shunt. Probably, succinic semialdehyde formed from GABA is converted to gamma-oxybutyric acid (GHB), which has a stronger inhibitory effect than GABA, the deficiency of which we found, and is able to limit the harmful effects of transmitter autocoidosis after PH (Wong et al., 2003; Jung et al., 2021). Thus, in the brain of offspring after PH inhibition of oxidative energy production, its transport and utilization, and activation of compensatory pathways of ATP formation - glycolysis and Roberts shunt - are observed, which, however, do not fully provide the brain energy demand and cause the development of lactate acidosis and deficiency of inhibitory amino acids. This determines the necessity of pharmacological correction of mitochondrial dysfunction. As it is known, the processes of mitochondrial dysfunction formation are significantly influenced by the deficiency of intra-mitochondrial HSP 70 revealed by us in early studies (Belenichev et al., 2023b; Belenichev et al., 2024a).

It is also shown that pharmacological positive mitochondrial dysfunction after PH is apparently one of the causes of oxidative stress activation in the neonatal brain due to hyperproduction of ROS in “parasitic” bioenergetic reactions (Hu and Zhang, 2021; Guo et al., 2024). Also, an oxidative stress occurs against the background of decreased expression of antioxidant enzymes (Kim et al., 2020; Singh et al., 2025). SOD and glutathione-dependent enzymes are of particular importance in brain protection after PH. Thus, GPX-4, capable of directly reducing the formation of hydroperoxides of phospholipids, fatty acids, cholesterol and thymine, exhibits a protective effect in relation to neuronal membranes. GPX-4 participates in endogenous antiaptic mechanisms, prevents the initiation of ferroptosis (Arai et al., 1999; Roveri et al., 1992). A very important aspect in the general biologic function of GPx4, as it has been found that PH can lead to ferroptosis of human trophoblast cells and then induce spontaneous abortion (Tian et al., 2024). GPx4 exhibits mitoprotective effect and inhibits the formation of mitochondrial dysfunction (Oh et al., 2022). GPx-1 reduces excessive cytotoxic concentration of H_2_O_2_ and inhibits H_2_O_2_-dependent mechanisms of formation of mitochondrial dysfunction and apoptosis against the background of thiol-disulfide system disorders, as well as plays an important role in preserving the bioavailability of NO (Lubos et al., 2011; Forgione et al., 2002; Belenichev et al., 2024d). Inhibition of GPx-1 and GPx-4 expression in the brain of offspring after PH may be directly related to nitrosative stress (Handy and Loscalzo, 2022; Peeples and Genaro-Mattos, 2022; Popazova et al., 2023). Cu/ZnSOD is localized in the cytoplasm, respectively, and serves as the main ROS sink in the intracellular environment and attracts much attention due to its physiological function and therapeutic potential, while its low concentration and activity is the cause of adverse outcomes after PH, as ROS excess have a detrimental effect on pregnancy and fetal development, disrupting the function of the placenta and impairing the delivery of oxygen and nutrients to the developing fetus and contributes to CNS disorders (Kong et al., 2017; Giles et al., 2002; Shim and Kim, 2013).

There is a close relationship between the level of glutathione and enzymes dependent on it, as well as the expression of HSP70, which provides a mechanism of endogenous neuroprotection, preservation of cognitive and mental functions of the CNS. Positive modulators of HSP70 are able to restore the glutathione link of the thiol-disulfide system and reduce the impairment of SH/SS-dependent intracellular signaling (Belenichev et al., 2023b; Zhang et al., 2022). The identified complex disorders after PH —including impaired energy metabolism, energy deficiency, dysfunction of the GABAergic system with reduced GABA levels, activation of nitrosative stress, suppression of early response gene expression (such as c-fos), initiation of neuroapoptosis, and downregulation of antioxidant enzyme expression—collectively contribute to delayed growth and neurodevelopment in the offspring. Our findings are consistent with previous research demonstrating that impairments in higher central nervous system functions after PH are associated with ATP depletion, glutamate-induced excitotoxicity, oxidative stress, and reduced gene expression activity (Sab et al., 2013; Robinson et al., 2005; Silvestro et al., 2020; Katche et al., 2010; Piña-Crespo et al., 2014; Villamena and Forman, 2025).

**Figure 3:**
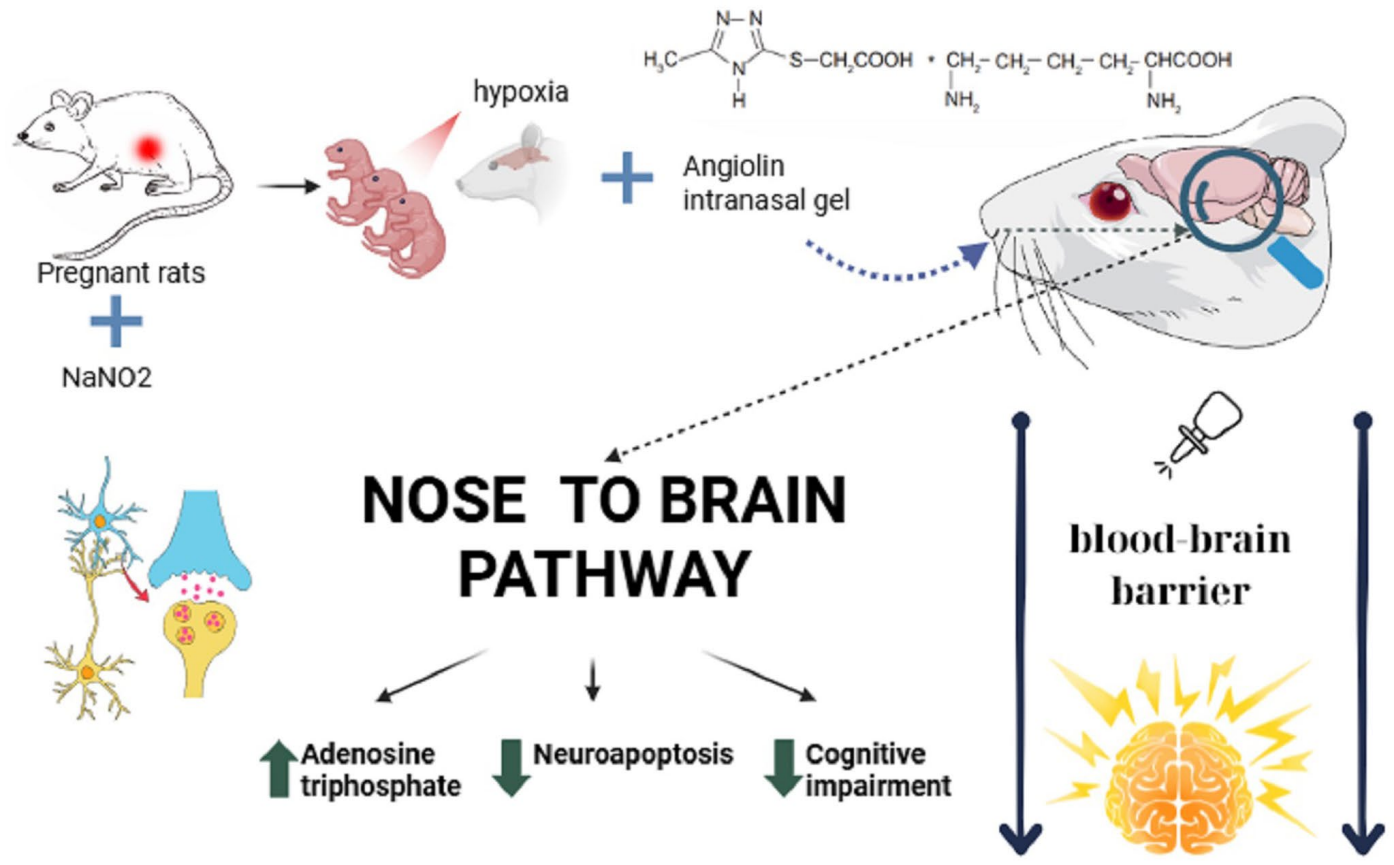
Mechanism of action of the Angiolin gel when administered intranasally.

The intranasal route of administration is promising for drug delivery directly to the central nervous system (CNS), bypassing the blood–brain barrier, which has traditionally been a difficult task for other dosage forms, which opens up new possibilities for achieving positive therapeutic effects in the treatment of neurological disorders, especially after perinatal CNS damage (Martinez et al., 2012; Crowe et al., 2018; Jeong et al., 2023; Qiu et al., 2025). The intranasal route of administration differs from other routes in its high bioavailability, lack of presystemic metabolism of active substances and potential use in neonatology (Snyers et al., 2022). The obtained results on the pharmacodynamics of neuroprotective action of Angiolin gel at intranasal administration after PH are in line with the views about the mechanism of action of this new original molecule (Figure 3). The revealed action of Angiolin gel on GABAergic system is connected with the ability of a fragment of its molecule L-lysine to transform into pipecolic acid, which increases affinity of GABA receptors and positively affects cAMP metabolism. This action of Angioltn provided functional equilibrium of excitation and inhibition processes in the brain of animals after PH and limited excitation of NMDA-receptors, thereby reducing the development of glutamate “excitotoxicity,” which leads to a decrease in neuropaptosis, preserves the density of neurons and glia in CA1 hippocampus, at the end leads to a decrease in disorders of cognitive-mnestic functions of the CNS in offspring after PH (Aliyeva et al., 2025; Belenichev et al., 2015a). This mechanism of Angiolin, namely, the reduction of excitotoxicity through increased affinity of GABA receptors, provides it with the properties of a primary neuroprotectant.

Secondary neuroprotection of Angiolin is associated with positive modulation of HSP70 and is aimed at reducing mitochondrial dysfunction and energy deficiency. The increase of HSP70 inside mitochondria and in the cytosol under the action of Angiolin leads to an increase in the lifetime of HIF-1, which ensures the activation and regulation of the most hypoxia-resistant energy shunt—malate–aspartate shunt (Belenichev et al., 2023b; Gogate et al., 2012; Bakleh and Al Haj Zen, 2025). Angiolin exhibits direct mitoprotective effect associated with HSP70 modulation - it reduces mitochondrial ultrastructural damage, regulates the rate of mitochondrial pore opening, preserves mitochondrial membrane potential (∆*Ψ*), inhibits the release of proapotic factors from the mitochondrion (Belenichev et al., 2016). Angiolin prevents the phenomena of lactate acidosis in the brain of newborns, which is a negative prognostic factor in PH (Belenichev et al., 2023a). By positively modulating HSP70, Angiolin may reduce cognitive impairment associated with the effects of PH. Since HSP70 deficiency may cause impairment of memory formation, its low concentration will impair the mechanisms of folding and transport of synaptic proteins that modulate signaling cascades associated with synaptic activation and participate in the mechanisms of neurotransmitter release (Singh et al., 2025; Kang et al., 2023). Angiolin also exhibits rather high antioxidant properties both due to its NO scavenger and due to increased activity of antioxidant enzymes (Belenichev et al., 2024c; Belenichev et al., 2024d). Piracetam had an insignificant positive effect on offspring development and higher CNS functions after PH. This is due to the peculiarity of its effect on brain energy metabolism in hypoxia and ischemia (Keil et al., 2006; He et al., 2008; Gouhie et al., 2024). Piracetam, increasing lactate production, increases the formation of lactate acidosis, which requires tighter control when it is used in neonatology (Al-Mosawi, 2020; Pressler and Mangum, 2013).

## 5 Conclusion

Preclinical studies using a prenatal hypoxia (PH) model demonstrated pronounced neuroprotective and nootropic activity of Angiolin gel when administered intranasally to experimental animals at a dose of 50 mg/kg during the first 30 days of postnatal life. Angiolin gel exerted a beneficial effect on both physical and cognitive development in neonates and contributed to a reduction in early neonatal mortality. It is well established that more than one-third of infants born after PH exhibit a high incidence of infectious, cardiovascular, and neuropsychiatric disorders, often accompanied by signs of polysystemic disorder of cellular energetics. One of the key pathogenic mechanisms in such cases is mitochondrial dysfunction. Hereditary (primary) mitochondrial dysfunctions associated with damage to the nuclear genome are known and described. Among them are various forms of infantile myopathies, Alpers disease, Leigh syndrome, Barth syndrome, Menkes disease, and deficiencies of carnitine or specific Krebs cycle and mitochondrial respiratory chain enzymes. Children after PH more frequently exhibit signs of secondary mitochondrial dysfunction, for which neuroprotective therapy is considered particularly effective, enabling significant clinical improvement across multiple domains of dysfunction. Angiolin is an original pharmacological agent with a unique combination of neuroprotective, anti-ischemic, and antioxidant properties. When administered intranasally, Angiolin gel demonstrated a favorable effect on the brains of neonatal rats after PH, promoting mitochondrial energy production and transport, reducing lactate acidosis, and decreasing the inefficient utilization of neurotransmitter amino acids in compensatory ATP-generating pathwaysAngiolin gel also normalized morphofunctional parameters of hippocampal neurons in offspring after PH, including reductions in the number of apoptotic and destructively altered cells and increased transcriptional and translational activity, as reflected by elevated RNA content and c-fos expression. The outcome of this experimental therapy included a reduction in neonatal mortality, as well as significant improvements in cognitive function and decrease in neurological disorders in the offspring. These findings are an experimental justification for further clinical trials of Angiolin gel (Angiolin with the permission of the State Expert Center of the Ministry of Health of Ukraine successfully passed the 1st stage of clinical trials), in order to introduce it into clinical practice of neurology and neonatology for treatment of PH consequences.

## Data Availability

The raw data supporting the conclusions of this article will be made available by the authors, without undue reservation.
